# Epidemiology and Clinical Outcomes of *Fusobacterium* Infections: A Six-Year Retrospective Study

**DOI:** 10.3390/medicina60020248

**Published:** 2024-01-31

**Authors:** Akram Khan, Hamza Alzghoul, Abdul Ahad Khan, Gopal Allada, Juliann M. Gronquist, Jonathan Pak, Srini Mukundan, Bishoy Zakhary, Raghav Wusirika, Nehan Sher, Raju Reddy

**Affiliations:** 1 Department of Internal Medicine, Oregon Health and Science University, Portland, OR 97239-3098, USAzakhary@ohsu.edu (B.Z.);; 2 Graduate Medical Education, College of Medicine, University of Central Florida, Orlando, FL 32827, USA; hamzehzghoolmd96@gmail.com; 3 Division of Pulmonary, Critical Care and Sleep Medicine, Banner University Medical Center, College of Medicine-Phoenix, University of Arizona, Phoenix, AZ 85721, USA; mirahadk@gmail.com (A.A.K.);; 4 Department of Nursing, Mirabella Portland, Portland, OR 97239, USA; 5 Knight Cardiovascular Institute, Oregon Health and Science University, Portland, OR 97239-3098, USA; mukundan@ohsu.edu

**Keywords:** *Fusobacterium* infections, *Fusobacterium* epidemiology, Lemierre syndrome, clinical outcomes, anaerobic bacteremia

## Abstract

*Background and Objectives*: Anaerobic bacteria like *Fusobacterium* can lead to severe and life-threatening infections. The inherent complexities in the isolation of these bacteria may result in diagnostic and therapeutic delays, thereby escalating both morbidity and mortality rates. We aimed to examine data from patients with infections due to *Fusobacterium* to gain insights into the epidemiology and clinical outcomes of patients with these infections. *Methods and Results*: We conducted a retrospective analysis of clinical data from a cohort of patients with cultures positive for Fusobacterium species at a tertiary care medical center in the United States. Between 2009 and 2015, we identified 96 patients with cultures positive for *Fusobacterium*. Patients could be categorized into three groups based on the site of primary infection. Patients with head and neck infections constituted 37% (n 36). Patients with infections of other soft tissue sites accounted for 38.5% (n 37). Patients with anaerobic bacteremia due to *Fusobacterium* formed 24% (n 23) of the cohort. Surgical intervention coupled with antibiotic therapy emerged as cornerstones of management for patients with head and neck or other soft tissue infections, who generally exhibited more favorable outcomes. Patients with bacteremia were older, more likely to have malignancy, and had a high mortality rate. When speciation was available, *Fusobacterium necrophorum* was the most frequently isolated species. *Conclusions*: Our retrospective analysis of epidemiology and clinical outcomes of *Fusobacterium* infections revealed three distinct cohorts. Patients with head, neck, or soft tissue infections had better outcomes than those with bacteremia. Our findings highlight the importance of employing management strategies based on infection site and underlying comorbidities in patients with *Fusobacterium* infections. Further research is needed to investigate the optimal therapeutic strategies and identify prognostic indicators to improve clinical outcomes for these complex infections.

## 1. Introduction

The *Fusobacterium* genus, comprising 13 species and part of the *Bacteroidaceae* family, is a critical player in human infections [1,2,3]. The most prevalent species implicated in human infections include *Fusobacterium necrophorum* (*F. necrophorum*)*, Fusobacterium nucleatum* (*F. nucleatum*), and *Fusobacterium varium* (*F. varium*). *Fusobacterium* is an obligate anaerobe characterized by its small spindle-shaped Gram-negative rod, non-spore-forming, and non-motile, which predominantly colonizes the oral, gastrointestinal, and upper respiratory tract [1,2,3]. Although these anaerobic bacteria are associated with infections, the extent of the infection often proves challenging to determine due to difficulties in effective microbiological identification and susceptibility tests [3,4,5]. *Fusobacterium* has been associated with Lemierre syndrome (LS), characterized by internal jugular vein thrombophlebitis [1,6,7]. In Lemierre syndrome, thrombophlebitis can occur in a variety of vessels in the head and neck, including the pharyngeal venous plexus and peritonsillar veins. LS has also been associated with cavernous sinus thrombosis and lateral sinus thrombosis.

Anaerobic bacteria like *Fusobacterium* are important causative pathogens for bacteremia and can lead to severe and life-threatening infections [8]. The difficulty isolating these bacteria can lead to delays in diagnosis and treatment, increasing morbidity and mortality. In a recent study, the incidence of bacteremia caused by *Fusobacterium* species was 64 per 100,000 in patients who underwent blood culture testing in 2020, which increased by 47.4% from 2011 to 2020 [8]. We performed a retrospective analysis of a cohort of subjects with *Fusobacterium*-positive cultures at a tertiary referral center in the Pacific Northwest of the USA to better define the epidemiology and clinical characteristics of Fusobacterium-associated infections.

## 2. Materials and Methods

After local institutional review board (IRB) approval, we retrospectively obtained a list of patients with cultures positive for *Fusobacterium* species at a tertiary care medical center in the Pacific Northwest between 2009 and 2015. Pre-reduced anaerobically sterilized plated and broth culture media were used for cultures based on institutional protocols. We obtained data from electronic health records to determine the clinical characteristics of patients, including demographics, comorbidities, clinical site of infection, duration of antibiotics, and disease course. We performed statistical analysis using STATA 16 (Stata Corp, College Station, TX, USA). A probability value of <0.05 was considered significant. Continuous variables were summarized using mean and standard deviation (±SD) and categorical variables with frequency (N) and relative frequency (%). We compared the clinical characteristics of survivors and non-survivors using chi-square tests for categorical variables and Wilcoxon tests for continuous variables. A one-way analysis of variance (ANOVA) was performed with a source of culture as the factor variable.

## 3. Results

We found that between 2009 and 2015, 96 subjects had positive cultures for *Fusobacterium species* (Table 1). A total of 63 (65.6%) of the cultures were not speciated; 28 (29.2%) were identified as *F. nucleatum*, 4 (4.2%) as *F. necrophorum,* and 1 (1%) as *F. varium*. Antibiotic susceptibility tests were not performed for culture-positive samples. While the incidence was higher in men compared to women, this was not statistically significant. The majority, 87.5%, of our subjects were Caucasian and 12.5% were of other races.

Infections were observed in the head and neck region in 36 (37.5%) patients, while 37 (38.5%) had infections in other soft tissues. A total of 23 (24%) patients had isolated bacteremia with positive blood cultures without an identified source of infection. This group included two patients with brain abscesses and one with septic thrombophlebitis without an identified focus of infection. Of the 96 patients, 23 (24%) had a malignancy, of which 13 were in the isolated bacteremia group (Table 1). The majority of the bacteremia patients, 11 (84.6%) had a hematological malignancy. A small percentage of patients in all 3 groups were immunosuppressed for other reasons such as solid organ transplant and immunosuppression for rheumatological disorders; however, there were no statistically significant differences between the three cohorts.

The mean age of our study population was 42.2 ± 22.2 years. A total of 15 (41.7%) of the patients in the head and neck group were 18 years old or younger compared to 4 (10.8%) in the soft tissue infection group and none in the isolated bacteremia group (Table 1). Patients with isolated bacteremia were significantly older than the other two study groups, with a mean age of 51.6 ± 16.7 years (Table 1). Of the 96 patients, 80 (83.3%) were hospitalized, while 16 (16.7%) were treated as outpatients with surgical treatment. A significant proportion of patients, 42 (43.8%), were hospitalized for more than seven days, and 26 (27%) required ICU admission (Table 2). Of the 96 patients, 89 (92.7%) received antibiotics. The other 7 (7.3%) who did not receive antibiotics were treated as outpatients with surgical treatment of the infection sites. Of the patients who received antibiotics, 52 (54%) received antibiotics for more than 14 days. Antibiotics used included metronidazole, penicillin G, amoxicillin-clavulanate, ampicillin-sulbactam, clindamycin, ceftriaxone, and piperacillin-tazobactam.

Most patients (78.1%) underwent surgical intervention (Table 2). Surgical interventions included abscess aspiration, incision and drainage, ear, nose, and throat surgery, and removal of surgical hardware. Surgical interventions were more common in patients with head and neck infections, 35 of 36 (97.2%). Furthermore, all 37 (100%) patients with infections in other soft tissue areas required surgical intervention. Compared to other groups, only 3 of 22 (13.6%) patients in the isolated bacteremia group required surgical intervention. In the isolated bacteremia group, two patients had brain abscesses drained, and one patient had a thrombectomy for septic thrombophlebitis. A total of 16 (16.7%) patients died during treatment. Mortality in the isolated bacteremia group was significantly higher at 43% compared to 8% in the other two groups (Table 2).

## 4. Discussion

Anaerobic bacteria like *Fusobacterium* can lead to severe and life-threatening infections. The inherent complexities in the isolation of these bacteria may result in diagnostic and therapeutic delays.

We conducted a 6-year retrospective study of culture-positive *Fusobacterium* infections at a tertiary referral center in the Pacific Northwest of the United States to gain insights into the epidemiology and clinical outcomes of patients with these infections. *Fusobacterium* infections are rare with incidence of bacteremia ranging from 0.53 to 64 per 100,000 cultures [5,8,9,10,11,12]. Our study is similar to a retrospective review that spanned 11 years (2000–2011) in the Calgary area of Alberta, Canada, which suggested an incidence of *Fusobacterium* bacteremia of 0.55/100,000 per year [5]. We found 23 patients with bacteremia in 6 years. Our data is similar to one retrospective series which identified 36 patients with bacteremia in 22 years and in a second series 26 patients over 10 years [9,13].

We found that patients with culture-positive *Fusobacterium* infections can be categorized into three broad groups. Patients with head and neck infections tend to be younger and have minimal comorbidities and low mortality. The second group includes patients with soft tissue infections of body parts other than the head and neck who tend to be older but have other characteristics similar to the first group. The third group of patients had isolated bacteremia without head, neck, or other soft tissue infection. This group of patients were older and had a high likelihood of malignancy, predominantly hematologic malignancy. This group also had a significantly higher mortality compared to the other two groups. A similar distribution was noted in three other studies including a cohort of patients with invasive *Fusobacterium* infections in the eight-year Swedish nationwide retrospective study, a five-year retrospective study from Taiwan, and a five-yearyear study in France [12,14,15].

Our data on patients with bacteremia is similar to other studies showing that the incidence of anaerobic bloodstream infections was significantly higher in older patients and those with malignancies, particularly hematological malignancies [4,16,17]. Previous observations of single institutions in Italy and Michigan hypothesized that severe mucositis was the highest risk of bacteremia in patients with underlying hematologic malignancy [17,18]. We did not notice a higher incidence of mucositis in our retrospective data analysis; however, our study focused on *Fusobacterium* infections, and our population may have differed from those studies. Our study showed a higher incidence of *F. nucleatum* bacteremia predominately in patients with malignancies, consistent with existing studies [3,19]. Of the 16 species typed samples in the bacteremia group, 15 were identified as *F. nucleatum*.

In 1936, Andre Lemierre published a series of 20 cases of throat infections with anaerobic septicemia, of which 18 patients died [20]. More recently, there has been a rapid increase in reporting of Lemierre syndrome [7]. According to a systematic review of data from 1950 to 2007, Lemierre syndrome was most commonly caused by *F. necrophorum* and only 3% were caused by *F. nucleatum* [21]. In our study, there was one case of confirmed Lemierre syndrome caused by *F. nucleatum*, a rare association.

We found that the majority of the patients with *Fusobacterium* infections need hospitalization with some needing the intensive care unit. This is similar to other studies that have shown a higher incidence of sepsis at presentation and the need for admission to the intensive care unit [9,12]. Similarly, our patients responded well to antibiotics. Patients required antibiotics for a prolonged duration; however, there were no differences between the three study groups.

In our study, 78% of the patients needed surgical intervention. All patients with miscellaneous soft tissue infections and almost all patients in the head and neck group (35 patients out of 36) underwent surgical intervention. On the contrary, in the isolated bacteremia group, only 3 patients of 23 had a surgical intervention. Two patients underwent CT-guided drainage of brain abscesses, and one patient underwent thrombectomy for jugular venous thrombosis. The mortality rate in our population was high at 16.7%. This is similar to another group that had a 10% mortality [5]. Other groups have shown lower mortality rates [9,12]. This difference in mortality between various studies may be related to the comorbidities of patients as well as the presence of sepsis. Like other studies, patients with isolated bacteremia had the highest mortality [5,9,12].

Our study has several limitations. We conducted a retrospective analysis in a single tertiary care center. *Fusobacterium* infections are rare and it would have been difficult to prospectively collect data. Our population was predominantly Caucasian and based in an urban setting and it may be difficult to generalize our data to the populations. In addition, similar to other studies, we did not have susceptibility testing or species-level identification for several of our cultures although these bacteria remain broadly susceptible to common antibiotics with limited utility of susceptibility testing [8,11]. It is also possible that there was an underestimation of outpatient cases, as some patients with soft tissue or head and neck infections may have received surgical interventions without culturing. In addition, we do not have longitudinal data on patient outcomes.

Despite these limitations, our study has several strengths, including a relatively large sample size allowing for generalizable conclusions in the population studied. We were able to divide our patients with *Fusobacterium* bacteremia into three different groups based on the sites of infection, which facilitated a more focused analysis of epidemiology, disease course, and treatment results for these groups.

Future research should focus on a few key areas to better understand *Fusobacterium* infections and improve patient outcomes. Firstly, more extensive studies should be initiated into the pathogenesis of *Fusobacterium* infections, especially in patients with bacteremia. Understanding how these bacteria trigger severe infections could lead to developing more effective therapeutic strategies, potentially reducing mortality rates. It would also be worthwhile to investigate the factors that make certain patients more susceptible to these infections, such as older age, malignancies, or other underlying comorbidities.

Secondly, research should also focus on improving the methods for isolation and identification of *Fusobacterium* species. The development of rapid and accurate diagnostic tools would enable early detection and intervention, thereby improving the prognosis for patients. Furthermore, given the high prevalence of *Fusobacterium* necrophorum, studies should also aim to better understand this species’s virulence factors, which could provide a foundation for developing targeted therapies or even a potential vaccine.

Lastly, given the significant impact of the infection site on patient outcomes, it would be beneficial to conduct studies to evaluate the efficacy of different management strategies based on the infection site. This could include comparing outcomes for patients with head and neck infections versus those with soft tissue infections or bacteremia. Similarly, research to identify prognostic indicators could improve patient management and outcomes. This could involve investigating the role of surgical intervention, antibiotic therapy, and patient characteristics such as age and underlying health conditions in determining the course of the infection and patient survival.

## 5. Conclusions

Analyzing a large group of patients with positive *Fusobacterium* cultures, we identified different groups based on the site of infection and characterized their associated risk factors, disease course, and response to treatment. Our study provides valuable information on the varied clinical spectrum and outcomes of *Fusobacterium* infections. We found that increased age and malignancy were significantly associated with *Fusobacterium* bacteremia, highlighting their importance in evaluating suspected cases. While *F. nucleatum* dominated the bacteremia group, supporting its growing role as a bloodstream pathogen, the classical Lemierre syndrome typically associated with *F. necrophorum* was rarely observed. Despite appropriate antibiotics and surgery, mortality remained high in patients with isolated bacteremia, underlining the need for better treatment strategies and prognostic markers. Surgical intervention was crucial in managing head, neck, and other soft tissue infections, highlighting its role alongside antibiotics for optimal results.

Our study emphasizes the diverse clinical spectrum of infections associated with *Fusobacterium*. It underscores the need for individualized patient management based on the site of infection, the underlying risk factors, and the identification of the species. More research is warranted to investigate optimal therapeutic strategies, elucidate the specific virulence factors of different species of *Fusobacterium*, and identify prognostic markers to improve clinical outcomes for these challenging infections.

## Figures and Tables

**Table 1:** Baseline Patient Demographics and Comorbidities.

Variable	Isolated Bacteremia (*n* = 23)	Head and Neck Infections (*n* = 36)	Miscellaneous Soft Tissue Infections (*n* = 37)	*p*-Value
Age at presentation (years)	51.6 ± 16.7	31.1 ± 22	47.1 ± 21.3	0.0003
Age 18 y or less (%)	0 (0%)	15 (41.7%)	4 (10.8%)	0.01
Height (m)	1.62 ± 0.10	1.62 ± 0.60	1.69 ± 0.16	0.22
Weight (kg)	64.14 ± 17.6	64.14 ± 29.0	81.8 ± 26.4	0.01
BMI (kg/m sq.)	27.7 ± 6.3	24.2 ± 6.4	27.6 ± 6.6	0.08
Gender Male/Female (*n* = 96)	15 (65%)/8(35%)	21 (58%)/15(42%)	23 (62%)/14(38%)	0.86
Race (White/Other) (*n* = 96)	22 (95.6%)/1 (4.3%)	30 83.3%)/6 (16.7%)	32 (86.5%)/5 (13.5%)	0.38
Immunosuppressed (*n* = 5)	1 (4.4%)	2 (5.6%)	2 (5.4%)	0.98
Malignancy (*n* = 23)	13 (56.5%)	3 (8.3%)	7 (18.9%)	<0.001

**Table 2:** Course of Disease and Microbial Characteristics.

Characteristics	Isolated Bacteremia (*n* = 23)	Head and Neck Infections (*n* = 36)	Miscellaneous Soft Tissue Infections (*n* = 37)	*p*-Value
Mortality (*n* = 16)	10 (43.5%)	3 (8.3%)	3 (8.1%)	0.0003
Hospitalized (*n* = 80)	22 (95.6%)	26 (72.2%)	32 (86.5%)	0.14
Length of stay >7 days, *n* = 42	15 (68.1%)	9 (34.6%)	18 (56.3%)	0.08
Length of stay ≤ 7days, *n* = 38	7 (31.9%)	17 (65.4%)	14 (43.7%)	
ICU stay (*n* = 26)	10 (43.5%)	7 (19.4%)	9 (24.3%)	0.24
Antibiotics > 14 days, *n* = 52	14 (63.6%)	18 (52.9%)	20 (60.6%)	0.81
Antibiotics ≤ 14 days, *n* = 37)	8 (36.4%)	16 (47.1%)	13 (39.4%)	
IV antibiotics alone (*n* = 28)	9 (39.1%)	10 (27.8%)	9 (24.3%)	0.62
Surgical intervention (*n* = 75)	3 (13%)	35 (97.2%)	37 (100%)	<0.001

## Data Availability

Data supporting the reported results are provided in the article. Additional deidentified data are available to the corresponding author.
